# Two Attentional Processes Subserving Working Memory Differentiate Gifted and Mainstream Students

**DOI:** 10.5334/joc.370

**Published:** 2024-05-23

**Authors:** Janice Johnson, Steven J. Howard, Juan Pascual-Leone

**Affiliations:** 1 Department of Psychology, York University, Toronto, CA; 2 School of Education, University of Wollongong, Wollongong, AU

**Keywords:** theory of constructive operators, mental attention, giftedness, perceptual attention, working memory, executive function

## Abstract

Two working memory (WM) measures were contrasted, to clarify the nature of advantages in gifted children’s cognitive processing. It was predicted that cognitively gifted children would excel in WM tasks taxing mental attention (i.e., n-back) but not tasks supported by perceptual attention (i.e., self-ordered pointing, SOPT). Ninety-one children aged 9–10 and 13–14 years, in a gifted or mainstream classroom, received n-back and SOPT, plus measures of mental-attentional (*M*-) capacity, inhibition, and shifting. Older children generally scored higher than younger children. As predicted, gifted children outperformed mainstream peers on all tasks, except for SOPT. Results demonstrate the need to distinguish between mental and perceptual attention in measurement of WM.

Conceptualisations of cognitive giftedness traditionally point to intellectual precocity (e.g., intelligence significantly exceeding the average for a given chronological age). Indeed, identification of children for inclusion in gifted education programs often is determined, at least in part, by performance on standardized intelligence tests. Research demonstrates that children identified as cognitively gifted are advantaged in numerous areas relative to their mainstream or average-ability peers, including: processing speed and efficiency (Aubry et al., 2021; Johnson et al., 2003), self-regulation (Calero et al., 2007), and executive functioning (Arffa, 2007; Johnson et al., 2003). Working memory (WM) has been implicated in performance on intelligence tests and, thereby, cognitive giftedness. Research shows a strong association between performance on WM and general ability measures in children and adults (e.g., Cowan et al., 2006; Pascual-Leone & Johnson, 2021; Vock & Holling, 2008), and high-ability children often demonstrate increased WM capacity compared with their average-ability peers (Alloway & Elsworth, 2012; Aubry et al., 2021; Johnson et al., 2003; Pascual-Leone & Johnson, 2011, 2021; Rodríguez-Naveiras et al., 2019).

WM refers to a capacity for maintaining information concurrently in mind, in an active state, for use in current processing. Yet measures of WM tap a range of processes: “encoding, maintenance, recall, recognition, familiarity, updating, temporal ordering, binding, attention, and inhibition” (Redick & Lindsey, 2013, p. 1110). Perhaps for this reason, association between WM measures (e.g., complex span tasks and n-back) often are weak (e.g., Kane et al., 2007; Redick & Lindsey, 2013). We use the Theory of Constructive Operators (TCO) (Pascual-Leone & Goodman, 1979; Pascual-Leone & Johnson, 2005, 2011; 2021) to distinguish between two WM measures and, in doing so, advance a theoretical distinction between mental attention and perceptual attention, processes that often are conflated in WM theorizing. Specifically, we examine the differences in performance between gifted and mainstream child samples across WM, mental attentional (*M*-) capacity, inhibition, and shifting tasks, with a particular interest in contrasting the n-back WM task (i.e., indicate if a stimulus is the same as the one presented *n* items earlier) with the self-ordered pointing task (SOPT; continually point to a new stimulus in an array that continues to spatially reorganise stimuli locations).

## Attentional Processes Subserving Working Memory: Mental Versus Automatic-Perceptual Attention

Current formulations of WM emphasize the link between WM and control mechanisms, such as attention and executive functions. Controlled attention is central in theories of WM, such as Cowan’s (Cowan, 2016) *scope of attention*, Engle’s (2010) *executive attention*, and Ruchkin and colleagues’ (Ruchkin et al., 2003) *attentional pointer system*. These formulations construe WM as the product of control mechanisms directing one’s limited attentional resources for the simultaneous, short-term activation of informational units in mind (e.g., chunks or schemes). Attention is a capacity-limited resource for boosting activation of these information-bearing units. Control mechanisms (or executive functions) serve to direct attention toward task-relevant information (e.g., shifting, updating) or away from task-irrelevant information (e.g., inhibition).

Neuroscientific evidence implicates distinct brain structures and processes involved in WM task performance. Foremost among these are the contributions of the dorsolateral prefrontal cortex and prefrontal dopamine contributing to activation, inhibition, and coordination of cortical pathways (Diamond et al., 1997, 2004; Petrides & Milner, 1982). There are inconsistencies, however, in the proposed involvement of prefrontal dopamine. Success on the SOPT (a WM task commonly used to assess prefrontal executive function) is similarly reliant upon the dorsolateral prefrontal cortex (Diamond et al., 2004; Petrides & Milner, 1982); however, SOPT performance appears unaffected by depleting prefrontal dopamine (Diamond et al., 1997, 2004; Petrides & Milner, 1982). Moreover, Brocki et al. (2008) compared children with attention-deficit/hyperactivity disorder (ADHD) and age-matched typically developing control children on verbal and visuo-spatial WM tasks. They found that children with ADHD, which is associated with comparatively lower dopamine levels, scored lower on the WM tasks, with the exception of SOPT. In contrast, performance on the n-back WM task has been found to relate to dopaminergic mechanisms (e.g., Aalto et al., 2005; Chatham et al., 2011; Goldberg et al., 2003).

An explanation for this discrepancy is found in the TCO’s distinction between effortless (often externally driven) automatic-perceptual attention and effortful, executive-driven mental attention (Arsalidou et al., 2013; Pascual-Leone & Johnson, 2021). The TCO hypothesizes that these distinct forms of attention are mediated by different neurotransmitters according to the functional characteristics of the task (Pascual-Leone & Johnson, 2021). Automatic-perceptual attention, which is posited as mediated by the acetylcholinergic neurotransmitter, is implicated in cognitive processing of often external information that is – and remains – perceptually available (Pascual-Leone & Johnson, 2021). In contrast, mental attention, believed to be mediated by the dopaminergic neurotransmitter, entails voluntary, often effortful, executive-driven processing of internal (mental) information (Pascual-Leone & Johnson, 2021). The TCO distinction between automatic-perceptual and mental attention is similar to that between the processing of externally versus internally generated information (Christoff & Gabrieli, 2000), between external versus internal attention (Chun et al., 2011), or between Type 1 and Type 2 thinking (Evans & Stanovich, 2013; Kahneman, 2011).

Mental attentional processing is particularly required in misleading situations, in which strongly activated information is incompatible with correct performance. This requires that task-compatible information be effortfully activated using mental attentional processes. This contrasts with facilitating situations, in which schemes activated by the context are congenial with correct performance (Pascual-Leone & Johnson, 2021).

## Linking Mental Attention, Perceptual Attention, Working Memory, and Cognitive Giftedness

According to the TCO, mental attention is comprised of: a limited capacity to effortfully activate schemes relevant for a given task (this is called mental or *M*-capacity); a resource for inhibiting schemes (*I*-operator) that interfere with correct task performance; and the executive schemes that are activated to guide application of *M* and *I*. Note that within the TCO, inhibition is considered to be a mental resource, not an executive function (Im-Bolter et al., 2015). *M*-capacity increases with age in normal child development and is positioned as a causal factor underlying developmental growth in WM (Pascual-Leone & Johnson, 2005, 2011; 2021). The capacity of *M* is predicted to grow by one unit every other year from 3 (1 unit) to 15 years of age (7 units), and empirical data support this growth function (e.g., Arsalidou et al., 2010; Pascual-Leone & Johnson, 2005, 2011; 2017; 2021). In contrast, automatic-perceptual attention (controlled by the brain’s default network) is believed to develop earlier and more rapidly than mental attention.

Consistent with their performance on WM and executive function tasks, gifted children tend to outperform their mainstream peers on measures of *M*-capacity (Johnson et al., 2003; Navarro et al., 2006; Pascual-Leone & Johnson, 2011, 2021). Although in administration of measures of *M*-capacity attempt is made to teach requisite task executives, control executives still are needed to coordinate application of *M* and *I*. Thus, high performance on *M*-measures could result from a heightened *M*-capacity, superior executive processes, or both, due to the concurrent requirement for activation and inhibition in these tasks (Pascual-Leone & Johnson, 2021). This is not unique to measures of *M*-capacity; WM tasks also vary in the extent to which they require executive control (Best & Miller, 2010; Luciana et al., 2005). In addition to demand for mental attentional capacity and need for executive control, we highlight the TCO’s distinction between a task’s requirement for primarily mental attentional versus automatic-perceptual attentional processing.

We sought to investigate this distinction by leveraging the known cognitive advantages of gifted over mainstream children. In addition to replicating the established advantage of gifted children on measures of *M*-capacity, inhibition, and executive function, we further predicted that children identified as gifted would score higher on a WM task requiring mental attention (i.e., n-back task), but not on a WM task that involved primarily automatic-perceptual attention (i.e., SOPT). We predict this based on evidence that gifted children possess superior executive know-how (and, for some, developmentally advanced *M*-capacity; Pascual-Leone & Johnson, 2021, ch. 7) – factors that would offer less advantage in facilitating situations requiring primarily perceptual attention (such as SOPT). For both the gifted and mainstream samples, we recruited children from two age groups (9–10 and 13–14 years). These ages were selected to cover two distinct developmental stages of *M-*capacity and to span the predicted *M*-capacity demands of the key tasks. This allowed test of our expectation that older children would score higher on all tasks, including SOPT (Archibald & Kerns, 1999; Cragg & Nation, 2007).

## Method

### Participants

Participants were 91 children at an elementary school in the Greater Toronto Area, drawn from one of four classes: grade 4 mainstream (*n* = 22), grade 4 gifted (*n* = 28), grade 8 mainstream (*n* = 22), and grade 8 gifted (*n* = 19). The gifted groups were in congregated classrooms for children identified as gifted. Identification as gifted required a minimum achievement of 97th percentile on standardized intelligence or ability tests. Children categorized as mainstream were in regular classrooms. The sample ranged in age from 9.28 to 14.23 years (grade 4: *M* = 9.81, *SD* = 0.33; grade 8: *M* = 13.72, *SD* = 0.31) with a balance of boys and girls (50.5% girls).

### Measures

All computer-based measures were programmed in E-Prime 1.2 (Psychology Software Tools, Pittsburgh, PA) and were presented on a laptop. Responses to the SOPT were made via touchscreen using a stylus. All other computerized tasks required keyboard button presses to respond.

#### N-back (WM Task Requiring Primarily Mental Attention)

N-back requires participants to continually update a set of stimuli held in mind, adding newly relevant stimuli at the expense of those that are no longer task-relevant (Cohen et al., 1997; Friedman et al., 2006; Kane et al., 2007; Im-Bolter et al., 2006). Updating of the contents of WM is required because participants are asked to evaluate whether the current stimulus matches one presented *n* items earlier, and which is no longer perceptually available (performance thus requires primarily mental attention). Updating often is classified as an executive function (e.g., Im-Bolter et al., 2015; Miyake et al., 2000). In the TCO, updating corresponds to an executive process of recentration, “a change in centration that changes content but not level of analysis” (Pascual-Leone & Johnson, 2021, p. 421).

We used a computer-based task with three levels that were presented in ascending order of difficulty. In the 0-back condition, participants identified whether each new stimulus matched a particular three-dot pattern. In the 1-back condition, they identified whether each new stimulus matched the immediately preceding three-dot pattern. In the 2-back condition, participants identified if each new stimulus matched the three-dot pattern presented two items earlier in the sequence.

Stimuli were nine distinct three-dot patterns presented one at a time, in a semi-random order. For each condition children received verbal training, 15 paper-based practice trials using cue cards (repeated up to three times if the child did not understand the task), 14 computer-based practice trials, and 54 task trials. On the computerized trials, participants indicated by a key press whether the current pattern matched the target pattern. Each stimulus was presented for 500 ms, followed by a 2500 ms gap during which the child could respond. A tone signalled responses that were incorrect or made beyond the trial time limit. The score was the proportion of correct target identifications on match trials. Reliability across the levels was good (α = .72).

#### SOPT (WM Task Requiring Primarily Perceptual Attention)

In the SOPT (Petrides & Milner, 1982), participants see an array of *n* stimuli and must point to a stimulus they have not previously pointed to. After each selection, the array reorganizes spatially and participants must select another new stimulus, continuing for a number of trials equal to the number of stimuli in the array (allowing each to be selected exactly once). We used the abstract condition of SOPT developed by Cragg and Nation (2007). Children were shown an array of abstract designs (in set sizes of 4, 6, 8, or 10 designs) and selected (via touch) any design to begin. After a selection, the next screen appeared with the same designs but in different locations (this perceptual availability meaning that performance is supported primarily by perceptual attention). Each set size was repeated three times. Designs within each set size remained constant (differing only in location) but differed between set sizes. Task levels were presented in order of ascending set size, with the first level (4 designs) used as training. Abstract designs were used due to difficulty of encoding them verbally, minimizing the possible influence of differential linguistic ability between groups. Performance was indicated as a span score; the mean number of correct touches until the first error within an array (removing the first trial, for which an error was not possible). Results were similar when data were analyzed as accuracy (proportion correct selections). Reliability of span scores across the three levels was acceptable (α = .66).

Although SOPT performance is supported by automatic, perceptual attention, it still carries a mental demand. A possible criticism is that this mental demand is too low to show a difference between gifted and mainstream students. To complexify the SOPT we developed a version that carried an additional executive demand for updating, while still preserving primary demand for perceptual attention. Only the 4, 6, and 8 design levels were administered in this U-SOPT, and once again, the 4-design level was used for training. In this variant, after selecting a new design, children had to then indicate also their just-previous selection. Performance was again indicated as a span score; the mean number of screens until an error in either selecting a new design or identifying the previously selected design (after removing the first trial, for which an error was not possible). Reliability of span scores across the levels was acceptable (α = .70).

#### M-Capacity

The Figural Intersections Task (FIT; Pascual-Leone & Baillargeon, 1994; Pascual-Leone & Johnson, 2011) is a paper-based measure of *M*-capacity. Each item depicts a set of two to eight discrete shapes on the right-hand side of the page, and the same set of shapes in an overlapping configuration on the left-hand side (for some items there is an additional irrelevant shape, to be ignored, on the overlapping configuration). Participants must locate the one area of common intersection of the relevant overlapping shapes on the left. Item levels are defined by the number of relevant shapes to be held in mind in order to find the intersection; this number also corresponds to the demand the item places on *M*-capacity (i.e., *M*-demand; Pascual-Leone & Baillargeon, 1994; Pascual-Leone & Johnson, 2011). The FIT was administered in a group session for each class, with each child independently completing their own booklet. FIT booklets consisted of 36 randomly ordered items (ranging from difficulty levels 2 to 8). There were five items at each difficulty level, except level 4 which had six items. Eight practice items preceded these test trials. Performance was indexed by an *M*-score, corresponding to the highest item level with at least 80% of items solved correctly, provided all lower levels also reached the 80% threshold (with one lower level permitted to fall to 60%). Reliability of the proportion of items correct across all levels was high (α = .81).

The Direction Following Task (DFT) is a verbal measure of *M*-capacity (Agostino et al., 2010; Cunning, 2003; Im-Bolter et al., 2006; Pascual-Leone & Johnson, 2011). Participants use foam cut-outs that vary in shape (circle or square), color (white, blue, green, red, or yellow), and size (small or large) to carry out verbal directions of increasing complexity. The directions ask participants to place a cut-out onto a space on a wooden board. Spaces vary in size and color. The task consisted of 35 items – five items at each of seven levels of complexity – preceded by verbal training and five practice items. Mental demand is a function of the number of cut-outs, spaces, and characteristics in the instruction (e.g., “Place a white square on a small blue space”, “Place a red square and a white circle on a small yellow space”). Directions referring to two cut-outs had to be carried out in the specified order. The cut-outs and board were covered while each instruction (item) was read aloud, after which the stimuli were made available to the subject to carry out the instruction. Score corresponds to the *M*-demand (Pascual-Leone & Johnson, 2011) of the highest level for which the child solved at least 60% of items correctly, provided all lower levels also reached the 60% threshold (with one lower level permitted to fall to 40%). Reliability of the proportion of items correct across all levels was high (α = .83).

#### Inhibition

The antisaccade task (adapted from Miyake et al., 2000) indexes inhibitory control (Agostino et al., 2010; Im-Bolter et al., 2006, 2015; Miyake et al., 2000). While focusing on a fixation point, participants are faced with a visual cue (0.4° – a solid black square) on one side of a computer screen, promptly followed by a target stimulus (2.0° – an arrow pointing up, right, or left inside a box) on the opposite side of the screen. Participants must inhibit their reflexive saccade toward the visual cue, instead looking toward the target stimulus (arrow) on the opposite side of the screen. Failure to inhibit this prepotent saccade would result in participants’ inability to accurately identify the arrow’s direction (indicated by pressing the ‘←’, ‘↑’, or ‘→’ key on the laptop keyboard). The timing of stimuli presentation was as follows: fixation cross for a variable time (1500–3500 ms); blank screen for 50 ms; cue for 225 ms; target for 100 ms, followed by a mask that remained on screen until a response was made. Twenty-two practice trials and 90 test trials were administered. The order of stimuli (arrow direction, left vs. right side of screen) was random for each subject. Performance on this task was indexed by the proportion correct target identifications.

#### Shifting

The Contingency Naming Task (CNT) was designed by Taylor et al. (1987) as a measure of cognitive flexibility (see also, Anderson et al., 2000). We used CNT to index the shifting executive function. In the TCO, shifting corresponds to an executive process of decentration, “a change of centration that changes both content and level of analysis” (Pascual-Leone & Johnson, 2021, p. 421). Participants were presented with a stimulus card that showed three rows of nine colored (blue, green, pink) shapes (square, circle, triangle), each enclosing an inner shape (square, circle, triangle). Above three stimuli in each row there was a backward pointing arrow. Participants were required to name aloud the color or outer shape of each stimulus, based on a set of rules. In the first trial, they named the color of each design, and in the second trial they named the outer shapes. The one-dimensional switching trial required naming the color when the inner and outer shapes matched or outer shape when there was no match. The two-dimensional shifting trial involved switching between two rules: 1) apply the rule from the one-dimensional switching task; but 2) reverse this rule in the presence of a backward arrow. Instructions emphasized speed and accuracy. Before each trial condition, children were told the relevant rule and practiced it on a seven-design practice card. Performance was indexed by an efficiency score that reflects both accuracy and speed, calculated as follows: [(1/time to complete the sub-task)/SQRT (errors + 1)] × 100. A higher score represents better ability to shift (Anderson et al., 2000). This score was computed for each of the four trials and for the total task using total time and total errors; the total score was used for correlations. Reliability of efficiency scores across levels was good (α = .77).

### Procedure

Tasks were administered in three sessions, two individual (in a separate, quiet classroom) and one group (in the students’ classroom), each lasting about 40 minutes. The order of task administration was held constant as follows: Individual Session 1 – SOPT, U-SOPT, CNT, DFT; Individual Session 2 – n-back, antisaccade; and Group Session – FIT. This study was performed in accordance with the Declaration of Helsinki and per the protocols approved by York University’s Research Ethics Board.

## Results

### Data Screening

Data first were screened to evaluate whether statistical assumptions were met. Because sphericity consistently was violated, adjusted degrees of freedom analysis (Greenhouse-Geisser) was conducted for all within-subjects effects. For all task scores in which outliers were present (i.e., n-back, DFT, and antisaccade accuracy) analyses were run with and without extreme observations. Because the pattern of results did not differ for any variable, we retained these observations in all reported analyses. One child was absent for the FIT group session. Table 1 contains descriptive statistics for all tasks. Partial correlations controlling for age appear in Table 2. There were numerous statistically significant correlations within the mainstream subsample, but few in the gifted sample.

**Table 1:** Mean Performance Scores as a Function of Grade and Academic Stream.


VARIABLE	GRADE 4		GRADE 8	
	
	MAINSTREAM M (SD)	GIFTED M (SD)	MAINSTREAM M (SD)	GIFTED M (SD)

Age	9.86 (0.38)	9.78 (0.30)	13.80 (0.26)	13.61 (0.33)

N-back				

0-back prop. correct	0.91 (0.08)	0.91 (0.08)	0.94 (0.07)	0.94 (0.06)

1-back prop. correct	0.72 (0.15)	0.81 (0.20)	0.78 (0.14)	0.83 (0.08)

2-back prop. correct	0.47 (0.15)	0.50 (0.15)	0.49 (0.21)	0.72 (0.21)

SOPT mean span	4.36 (0.82)	4.46 (0.86)	4.91 (1.00)	5.06 (0.82)

6 stimuli	3.65 (0.87)	3.80 (0.90)	4.09 (0.73)	4.16 (0.64)

8 stimuli	4.44 (1.29)	4.56 (1.35)	4.86 (1.29)	5.14 (1.23)

10 stimuli	5.00 (1.37)	5.01 (1.63)	5.79 (1.92)	5.89 (1.51)

U-SOPT mean span	2.61 (1.09)	3.08 (1.02)	3.98 (1.22)	3.98 (1.18)

6 stimuli	2.14 (1.02)	2.65 (1.10)	3.27 (1.15)	3.37 (1.17)

8 stimuli	3.09 (1.55)	3.50 (1.46)	4.68 (1.41)	4.60 (1.68)

FIT *M*-score	4.36 (1.18)	5.43 (1.40)	6.14 (1.56)	6.89 (0.94)

DFT *M*-score	4.59 (0.73)	5.18 (0.77)	5.36 (0.85)	6.16 (1.30)

CNT efficiency	0.41 (0.25)	0.44 (0.18)	0.48 (0.22)	0.73 (0.21)

Trial 1	4.84 (1.03)	5.23 (0.91)	6.65 (0.78)	6.75 (1.02)

Trial 2	4.24 (1.08)	4.27 (0.94)	5.50 (1.06)	5.97 (1.00)

Trial 3	1.70 (0.60)	1.74 (0.54)	1.92 (0.66)	2.71 (0.67)

Trial 4	1.08 (0.65)	1.18 (0.45)	1.36 (0.69)	2.01 (0.55)

Antisaccade prop. correct	0.68 (0.15)	0.74 (0.14)	0.79 (0.18)	0.89 (0.08)


*Note*: 0-back = 0-back level of the n-back Task; 1-back = 1-back level of the n-back Task; 2-back = 2-back level of the n-back Task; SOPT = Self-Ordered Pointing Task; U-SOPT = Updating Self-Ordered Pointing Task; FIT = Figural Intersections Task; DFT = Direction Following Task; CNT = Contingency Naming Task.

**Table 2:** Correlations between Tasks as a Function of Academic Stream, with Age Partialled.


		1	2	3	4	5	6	7	8

1.	FIT *M*-Score	**–**	.37*	.92**	.37*	.54**	.35*	.40**	.27

2.	DFT *M*-score	.14	**–**	.71**	.29	.40**	.41**	.42**	.30

3.	Mean *M*-score	.81**	.70**	**–**	.41**	.58**	.45**	.48**	.33*

4.	2-back prop. correct	.15	.21	.23	**–**	.36*	.08	.38*	.46**

5.	SOPT span score	.21	-.04	.13	.15	**–**	.38*	.41**	.16

6.	U-SOPT span score	.21	.20	.27	.19	.27	**–**	.32*	.10

7.	CNT total efficiency	.23	.09	.22	.02	.08	.29*	**–**	.33*

8.	Antisaccade prop. correct	.21	-.13	.08	.28	.23	-.02	.10	**–**


*Note*: Correlations coefficients for mainstream sample (*n* = 44) are presented above the diagonal, and coefficients for gifted sample (*n* = 47) are presented below the diagonal. FIT = Figural Intersections Test; DFT = Direction Following Test; Mean *M*-score = average of FIT and DFT; 2-back = 2-back level of the n-back Task; SOPT = Self-Ordered Pointing Task; U-SOPT = Updating Self-Ordered Pointing Task; CNT = Contingency Naming Task. *p < .05. **p < .01.

#### N-Back

A Greenhouse-Geisser (G-G) 2 (stream) × 2 (grade) × 3 (condition) ANOVA on proportion of correct responses on trials in which there was a match between stimulus and target (i.e., correct target identification) showed main effects for: academic stream, *F*(1, 87) = 8.81, *p* = .004, partial η^2^ = .09; grade, *F*(1, 87) = 7.86, *p* = .006, partial η^2^ = .08; and n-back condition, *F*(1.77, 154.07) = 238.02, *p* < .001, partial η^2^ = .73. Overall, gifted students (*M* = 0.78, *SD* = 0.11) scored higher than their mainstream peers (*M* = 0.72, *SD* = 0.11), grade 8s (*M* = 0.78, *SD* = 0.10) scored higher than grade 4s (*M* = 0.72, *SD* = 0.11), and scores decreased with each increase in n-back level (0-back: *M* = 0.93, *SD* = 0.08; 1-back: *M* = 0.78, *SD* = 0.16; 2-back: *M* = 0.54, *SD* = 0.19).

**Figure 1:**
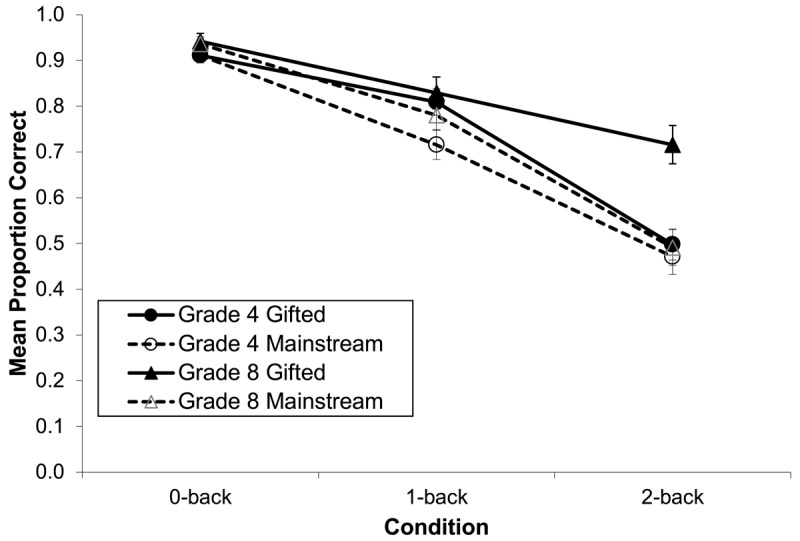
N-back mean proportion correct target identifications, as a function of grade, academic stream, and n-back condition. Error bars represent standard errors.

There were Stream × Condition, *F*(1.77, 154.07) = 6.16, *p* = .004, partial η^2^ = .07, as well as Grade × Condition interactions, *F*(1.77, 154.07) = 3.84, *p* = .028, partial η^2^ = .04. These were conditioned by a Stream × Grade × Condition interaction, *F*(1.77, 154.07) = 6.46, *p* = .003, η^2^ = .07. This interaction (see Figure 1) was examined using a 2 (stream) × 2 (grade) ANOVA within each n-back condition, and can be understood as follows: 1) There was no difference by stream or grade on the 0-back task (performance was uniformly high); 2) gifted children in both grades showed an advantage on the 1-back task; and 3) there was a gifted advantage only for the older children on the 2-back task— mainstream grade 8s and both grade 4 groups performed at chance levels on this task.

#### Self-Ordered Pointing Task (SOPT)

A G-G 2 (stream) × 2 (grade) × 3 (set size) ANOVA showed that: Grade 8s had longer spans than grade 4s, *F*(1, 87) = 9.73, *p* = .002, partial η^2^ = .10 (grade 4: *M* = 4.41, *SD* = 0.89; grade 8: *M* = 4.96, *SD* = 0.88); and span increased as set size increased, *F*(1.76, 153.33) = 37.92, *p* < .001, partial η^2^ = .30 (6 designs: *M* = 3.91, *SD* = 0.81; 8 designs: *M* = 4.71, *SD* = 1.30; 10 designs: *M* = 5.35, *SD* = 1.67). Gifted and mainstream children did not differ, however, in SOPT span score, *F*(1, 87) = 0.43, *p* = .516, partial η^2^ = .005 (Mainstream: *M* = 4.61, *SD* = 0.88; Gifted: *M* = 4.76, *SD* = 0.90). A Bayesian independent samples *t*-test, with JASP default priors, yielded B_01_ = 4.27, providing moderate support for the null hypothesis. Note that there also were no effects involving academic stream in mean number of correct responses (*p*s > .05).

#### Updating Self-Ordered Pointing Task (U-SOPT)

A G-G 2 (stream) × 2 (grade) × 2 (set size) ANOVA showed results similar to the SOPT: grade 8s outperformed grade 4s, *F*(1, 87) = 22.88, *p* < .001, partial η^2^ = .21 (grade 4: *M* = 2.85, *SD* = 1.14; grade 8: *M* = 3.94, *SD* = 1.12); and span increased with the number of designs, *F*(1, 87) = 53.63, *p* < .001, partial η^2^ = .38 (6 designs: *M* = 2.82, *SD* = 1.19; 8 designs: *M* = 3.90, *SD* = 1.64). However, gifted and mainstream children did not differ in terms of span score on U-SOPT, *F*(1, 87) = 0.98, *p* = .326, partial η^2^ = .01 (Mainstream: *M* = 3.26, *SD* = 1.11; Gifted: *M* = 3.53, *SD* = 1.15). A Bayesian analysis, using JASP default priors, yielded B_01_ = 3.95, supporting the finding of no difference between gifted and mainstream samples. Note that there were no effects involving academic stream (*p*s > .05) when span scores were analyzed separately for the correct selection of a previously unselected (‘new’) stimulus and when data were analyzed for mean number of correct old or new touches.

#### M-Measures

A 2 (stream) × 2 (grade) ANOVA on FIT *M*-score yielded main effects for stream, *F*(1, 86) = 10.70, *p* = .002, partial η^2^ = .11, and grade, *F*(1, 86) = 34.13, *p* < .001, partial η^2^ = .28. Gifted students (*M* = 6.02, *SD* = 1.42) scored higher than their mainstream peers (*M* = 5.23, *SD* = 1.63), and grade 8s (*M* = 6.50, *SD* = 1.34) scored higher than grade 4s (*M* = 4.96, *SD* = 1.40). Further, performance corresponded closely to theoretically predicted values (Pascual-Leone & Johnson, 2011, 2021); grade 4 mainstream children (aged 9–10) obtained mean *M*-scores close to four, and grade 8 mainstream children (aged 13–14) obtained *M*-scores close to six. Gifted children, in contrast, scored approximately one level higher than their mainstream counterparts (i.e., about five for grade 4s and about seven for grade 8s).

A 2 (stream) × 2 (grade) ANOVA run on the Direction Following Task (DFT) *M*-score yielded main effects for stream, *F*(1, 87) = 12.69, *p* = .001, partial η^2^ = .13, and grade, *F*(1, 87) = 20.40, *p* < .001, partial η^2^ = .19. Gifted students (*M* = 5.58, *SD* = 1.12) scored higher than their mainstream peers (*M* = 4.98, *SD* = 0.87), and grade 8s (*M* = 5.71, *SD* = 1.13) scored higher than grade 4s (*M* = 4.92, *SD* = 0.80). Performance level mirrored theoretical prediction for grade 4 mainstream students, but grade 8 mainstream students tended to underperform on the DFT relative to FIT and theoretical predictions. This pattern of results was maintained performing these same analyses on mean of *M*-scores across the two *M*-measures.

#### Inhibition

Proportion correct target identifications on the antisaccade task was examined with a 2 (stream) × 2 (grade) ANOVA. It yielded main effects for stream, *F*(1, 87) = 7.11, *p* = .009, partial η^2^ = .08, and grade, *F*(1, 87) = 18.93, *p* < .001, partial η^2^ = .18. Gifted children (*M* = 0.80, *SD* = 0.14) were more accurate than mainstream children (*M* = 0.73, *SD* = 0.16), and grade 8s (*M* = 0.84, *SD* = 0.15) were more accurate than grade 4s (*M* = 0.71, *SD* = 0.15).

#### Shifting

**Figure 2:**
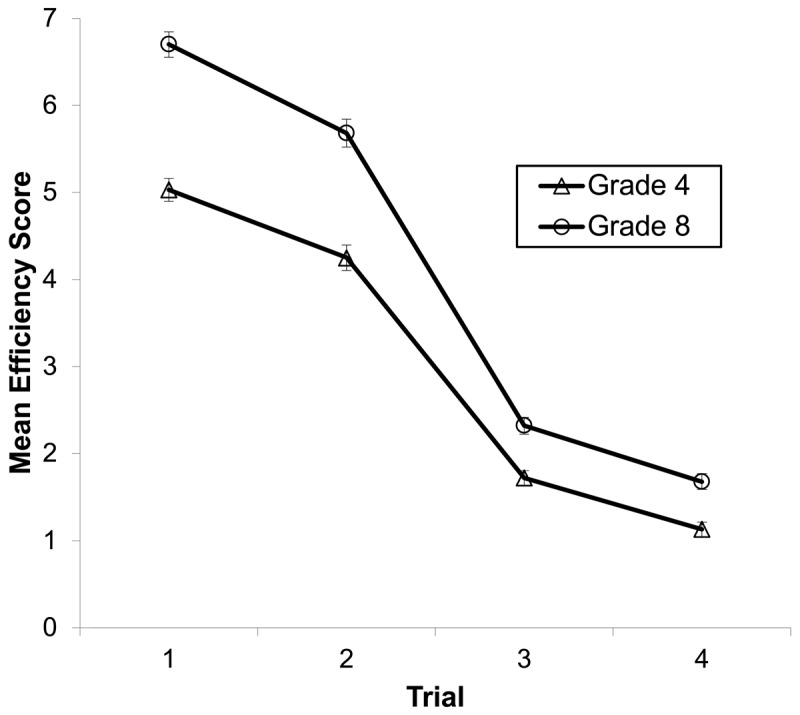
Contingency Naming Task (CNT) efficiency score, as a function of grade and CNT trial. Error bars represent standard errors.

A G-G 2 (stream) × 2 (grade) × 4 (trial) ANOVA on the CNT yielded main effects for: stream, *F*(1, 87) = 8.50, *p* = .005, partial η^2^ = .09; grade, *F*(1, 87) = 85.02, *p* < .001, partial η^2^ = .49; and trial, *F*(2.31, 203.51) = 919.74, *p* < .001, partial η^2^ = .91. Efficiency scores were higher for gifted (*M* = 3.61, *SD* = 0.80) than mainstream (*M* = 3.41, *SD* = 0.72), higher for grade 8s (*M* = 4.07, *SD* = 0.54) than grade 4s (*M* = 3.04, *SD* = 0.60), and decreased across the four CNT trials (trial 1: *M* = 5.80, *SD* = 1.24; trial 2: *M* = 4.90, *SD* = 1.24; trial 3: *M* = 1.97, *SD* = 0.71; trial 4: *M* = 1.37, *SD* = 0.67). These main effects were conditioned by a Grade × Trial interaction, *F*(2.31, 203.51) = 15.80, *p* < .001, partial η^2^ = .15. Grade 8s scored higher than grade 4s in all conditions; however, the difference was greater for speeded naming trials (trials 1 and 2) than for switching trials (trials 3 and 4; see Figure 2).

In sum, grade 8 students performed better than grade 4s on all tasks except the n-back, where grade 8s scored higher only for gifted children in the 2-back condition. Gifted students generally scored higher than mainstream peers, with the exception of the SOPT and U-SOPT tasks.

## Discussion

The current study sought to replicate well-documented cognitive advantages for gifted children on measures of mental attention (Johnson et al., 2003; Navarro et al., 2006; Pascual-Leone & Johnson, 2011, 2021), WM (Alloway & Ellsworth, 2012; Calero et al., 2007), and inhibition and shifting (Arffa, 2007; Johnson et al., 2003). Our novel prediction was that no gifted advantage would be found on a WM task that required primarily perceptual attention. This was based on our expectation that gifted children’s superior performance on measures of *M*-capacity, inhibition, shifting, and (at least some) WM tasks was related mainly to a richer executive repertoire, which would confer little benefit on a facilitating task requiring primarily perceptual attention. Consistent with these predictions, gifted children scored higher than mainstream peers on the n-back WM task, as well as measures of *M*-capacity, inhibition, and cognitive flexibility. They did not score higher on the SOPT, however, even when it was made more demanding by requiring that children point to both a new pattern and the just-previous one.

These findings support the TCO’s distinction between mental attention and perceptual attention. The n-back task requires matching stimuli that must be represented mentally, because they are presented and removed in quick succession. The presence of lures – matching stimuli that repeat in positions other than the criterion one (e.g., 3- or 4-back when the task is to match 2-back) – makes the n-back also a misleading task (Pascual-Leone & Johnson, 2021; Shipstead et al., 2016). Recent lures, in particular, may remain highly active in memory and lead to errors if not inhibited. In contrast, we proposed that SOPT engages primarily automatic-perceptual attention, because relevant stimuli remain available in the field of perception throughout the task. The task also is facilitating, without strongly misleading aspects (Pascual-Leone & Johnson, 2021). Given that older children scored higher than younger on both SOPT versions, failure to find an effect of academic stream was not due to a ceiling effect.

Task complexity (the expected *M*-demand) moderated the gifted advantage on the n-back. We found no gifted advantage on the 0-back task, which required matching the current stimulus to a criterion pattern. Gifted students in both grades were more accurate on the 1-back, but only grade 8 gifted were more accurate on the 2-back. Task analysis (see Pascual-Leone & Johnson, 2021, ch.10) of the 2-back task estimates its initial *M*-demand as 5 or 6 schemes that must be simultaneously activated by *M*-capacity. This exceeds the predicted the *M*-capacity of grade 4 children, and thus poor 2-back performance by the children in grade 4 is to be expected. The estimated *M*-demand of the 2-back task was within the theoretical capacity of grade 8s, however, and here a gifted advantage emerged.

The SOPT is also considered a WM task (Cragg & Nation, 2007; Diamond et al., 2004; Petrides & Milner, 1982; Ross et al., 2007), although Morra et al. (2018) have questioned this. While performance on SOPT can benefit from the use of *M*-capacity, performance is supported also by automatic-perceptual attention. Pascual-Leone and Johnson (2021, ch. 10) argued that there are two compatible strategies for SOPT. One relies on a feeling of familiarity (point to an unfamiliar design). The other uses mental attention but does not require inhibition of misleading aspects. Pascual-Leone and Johnson’s (2021) task analysis estimated a basic *M*-demand of 4 for SOPT, making it similar to 1-back in *M*-demand. Our results suggest that children identified as gifted excel at WM tasks that carry demand for mental attention in misleading situations, but not those that are facilitating and primarily engage perceptual attention.

Although these findings are consistent with the proposal that different attentional resources underlie performance on the n-back and SOPT, it also could be argued that results are a product of different *M*- or executive demands (n-back requiring participants to update the current attentional focus/centration). To address this issue, we designed the U-SOPT, which increased the *M*- and executive demand of the SOPT, by including an updating component. Crucially, the U-SOPT retained perceptual availability of stimuli within a facilitating situation. Consistent with TCO’s predictions, there was no gifted advantage on any U-SOPT score. These results provide further support for a distinction between mental attention and automatic-perceptual attention that can be less easily ascribed to other task characteristics.

Conceptions of WM typically consider all schemes (or chunks) within the individual’s repertoire that are sufficiently highly activated to affect the current performance. These approaches generally do not identify multiple sources for activation of schemes in WM. Cowan (2005), for example, considers schemes within the focus of attention, without identifying activation sources. Engle (e.g., Shipstead et al., 2016) emphasizes both the maintenance of relevant information and disengagement from (inhibition of) no-longer-relevant information. Mental attention, as defined by the TCO, is analogous to these constructs (Pascual-Leone, 1970; Pascual-Leone & Johnson, 2011, 2021). However, within the TCO, analysis of tasks includes all schemes that come to bear on performance and, uniquely, their distinct sources of activation (Pascual-Leone & Johnson, 2005, 2011; 2021). Such analyses have led to the claim that misleading situations are most suitable for measurement of mental attention (Arsalidou et al., 2010; Pascual-Leone & Johnson, 2005, 2011; 2021), because they require the control of task-irrelevant schemes that are activated by aspects of the context.

The current results cannot disentangle whether the gifted advantage on *M*-capacity, WM, inhibition, and shifting measures is a consequence of superior endogenous *M*-capacity, a more sophisticated repertoire of executive schemes, or both. The general absence of correlations of *M*-scores with other tasks in the gifted sample suggests, however, that rather than relying on an advanced endogenous *M*-capacity or general executive know-how, gifted children may rely on more specialized (task-specific) executive skills. The gifted students may have acquired a rich repertoire of schemes for dealing with specific kinds of tasks and relied on these schemes for their enhanced performance. In contrast, performance was generally correlated across tasks in the mainstream sample; in this sample an *M*-capacity score, corresponding to the average of scores on the two *M*-measures, correlated with scores on all other tasks. This suggests that mainstream students were relying more on general capacities and less on specific executive skills. This interpretation is consistent with EEG data reported by Gevins and Smith (2000), indicating that high ability subjects were more likely to develop task-specific strategies on the n-back task.

Lack of inter-task correlations in the gifted sample could arise from reduced variance, as this is a more homogenous (at least in IQ) group. However, examination of standard deviations suggests a similar degree of variance in performance of gifted and mainstream children across the range of tasks. Others have similarly noted smaller correlations in high-ability samples as compared with average-ability samples (e.g., Schofield & Ashman, 1986), even in cases where restricted range on the measures being correlated appeared not to be a problem (e.g., Redick & Lindsey, 2013).

## Conclusion

In conclusion, results of this study lend support to the TCO’s distinction between mental and automatic-perceptual attention as an important organismic factor in the measurement of WM. Differential performance patterns between gifted and mainstream students suggest that children identified as cognitively gifted (i.e., high ability children) excel at executive processing and at mobilizing and applying mental attention within mentally demanding, misleading situations. They do not excel, however, in facilitating situations where WM performance is supported by perceptual attention, even when these situations carry equivalent mental demand. This has implications for the measurement and theorizing of WM. Whereas differences in performance across WM tasks is often ascribed to differences in task characteristics, the current study’s empirical support for the TCO predictions suggests that, at least in some cases, these differences may arise from the different attentional processes underpinning performance.

## Data Accessibility Statement

The data that support the findings of this study are openly available in the York University Dataverse at https://doi.org/10.5683/SP3/ZK7F38.
